# Acylation-coupled lipophilic induction of polarisation (Acyl-cLIP): a universal assay for lipid transferase and hydrolase enzymes†
†Electronic supplementary information (ESI) available: Experimental materials and methods, additional Acyl-cLIP data, chemical structures, hit compound counter-screens, peptide sequences and characterisation. See DOI: 10.1039/c9sc01785b


**DOI:** 10.1039/c9sc01785b

**Published:** 2019-07-01

**Authors:** Thomas Lanyon-Hogg, Markus Ritzefeld, Lea Sefer, Jasmine K. Bickel, Amalie F. Rudolf, Nattawadee Panyain, Ganka Bineva-Todd, Cory A. Ocasio, Nicola O'Reilly, Christian Siebold, Anthony I. Magee, Edward W. Tate

**Affiliations:** a Department of Chemistry , Imperial College London , London , W12 0BZ , UK . Email: t.lanyon-hogg@imperial.ac.uk ; Email: e.tate@imperial.ac.uk ; Tel: +44 0207 5943752 ; Tel: +44 0207 5945821; b Division of Structural Biology , Wellcome Centre for Human Genetics , University of Oxford , Oxford , OX3 7BN , UK; c The Francis Crick Institute , London NW1 1AT , UK; d Molecular Medicine Section , National Heart & Lung Institute , Imperial College London , London , SW7 2AZ , UK

## Abstract

Posttranslational attachment of lipids to proteins is important for many cellular functions, and the enzymes responsible for these modifications are implicated in many diseases, from cancer to neurodegeneration. Lipid transferases and hydrolases are increasingly tractable therapeutic targets, but present unique challenges for high-throughput biochemical enzyme assays which hinder development of new inhibitors. We present Acylation-coupled Lipophilic Induction of Polarisation (Acyl-cLIP) as the first universally applicable biochemical lipidation assay, exploiting the hydrophobic nature of lipidated peptides to drive a polarised fluorescence readout. Acyl-cLIP allows sensitive, accurate, real-time measurement of *S*- or *N*-palmitoylation, *N*-myristoylation, *S*-farnesylation or *S*-geranylgeranylation. Furthermore, it is applicable to transfer and hydrolysis reactions, and we demonstrate its extension to a high-throughput screening format. We anticipate that Acyl-cLIP will greatly expedite future drug discovery efforts against these challenging targets.

## Abstract 2

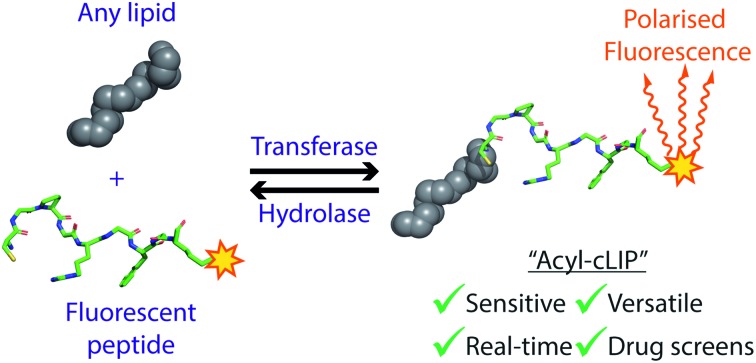
A highly accurate and versatile fluorescence polarisation assay for any enzyme adding or removing lipid posttranslational modifications, with the potential to accelerate drug discovery against these targets.

## Introduction

Posttranslational modification (PTM) of proteins with lipids is integral to many cellular processes, and dysregulation is implicated in diseases including cancer and neurodegeneration.^1^,^2^ Lipid PTMs include fatty acids and isoprenyl groups, and the enzymes responsible for their addition or removal are of great interest as therapeutic targets.^1^,^2^ Recent structural elucidation of key protein families involved in lipid PTMs^3^–^5^ along with development of powerful chemoproteomic methods to assess lipidation in living cells^6^–^8^ have highlighted their tractability as drug targets, for example, validating fatty acid transferases as therapeutic targets in malaria and in viral infections.^9^,^10^

Despite this progress, current biochemical assays to measure the activity of target enzymes present limitations that hinder drug discovery efforts. Classical methods for analysing lipidation use radioisotope-labelled lipids;^11^ however, such approaches are both hazardous and expensive. Fluorogenic methods have been developed through detection of by-products from lipidation reactions, such as Coenzyme A (CoA),^9^,^10^ although these may be susceptible to assay interference, and lack universality across classes of lipid PTM. In recent years, alkyne-tagged fatty acids have been used in ELISA-based formats;^12^,^13^ however, the multiple liquid handling steps limit throughput and introduce unnecessary complexity. Attachment of isoprenyl lipids has been studied using fluorogenic dansyl-RAS substrate peptides,^14^ but the structural requirements for fluorogenic behaviour can limit application to other lipid PTMs. Removal of fatty acyl modifications by hydrolase enzymes may be monitored through use of fluorogenic probes,^15^–^17^ although these probes are not applicable to lipid transferases.

To address the pressing and unmet need for a facile, versatile and robust assay for lipidation, we sought to develop a fluorescence polarisation assay driven by the increase in hydrophobicity upon lipidation which is universally applicable to all lipid PTMs. Fluorescence polarisation assays are commonly used to measure small fluorescent molecules or peptides binding to larger macromolecules, where binding results in a decrease in molecular tumbling and an increase in polarised fluorescence emission. Lipid PTMs are not macromolecular modifications, but we hypothesised that the increase in hydrophobicity could be used to bind the lipidated peptide to lipophilic macromolecular structures, resulting in decreased tumbling and increased polarisation.

Here, we report the development of a facile, versatile and robust assay for fatty acylation, which we term Acylation-coupled Lipophilic Induction of Polarisation (Acyl-cLIP). We demonstrate that this method is readily applicable to all lipid PTMs, and overcomes the limitations of all existing biochemical lipidation assays. Acyl-cLIP displays excellent characteristics for high-throughput screening, and provides a powerful method for future investigations and drug discovery programs against this important target class.

## Results and discussion

### Increased hydrophobicity from lipidation allows fluorescence polarisation readout

**Fig. 1:**
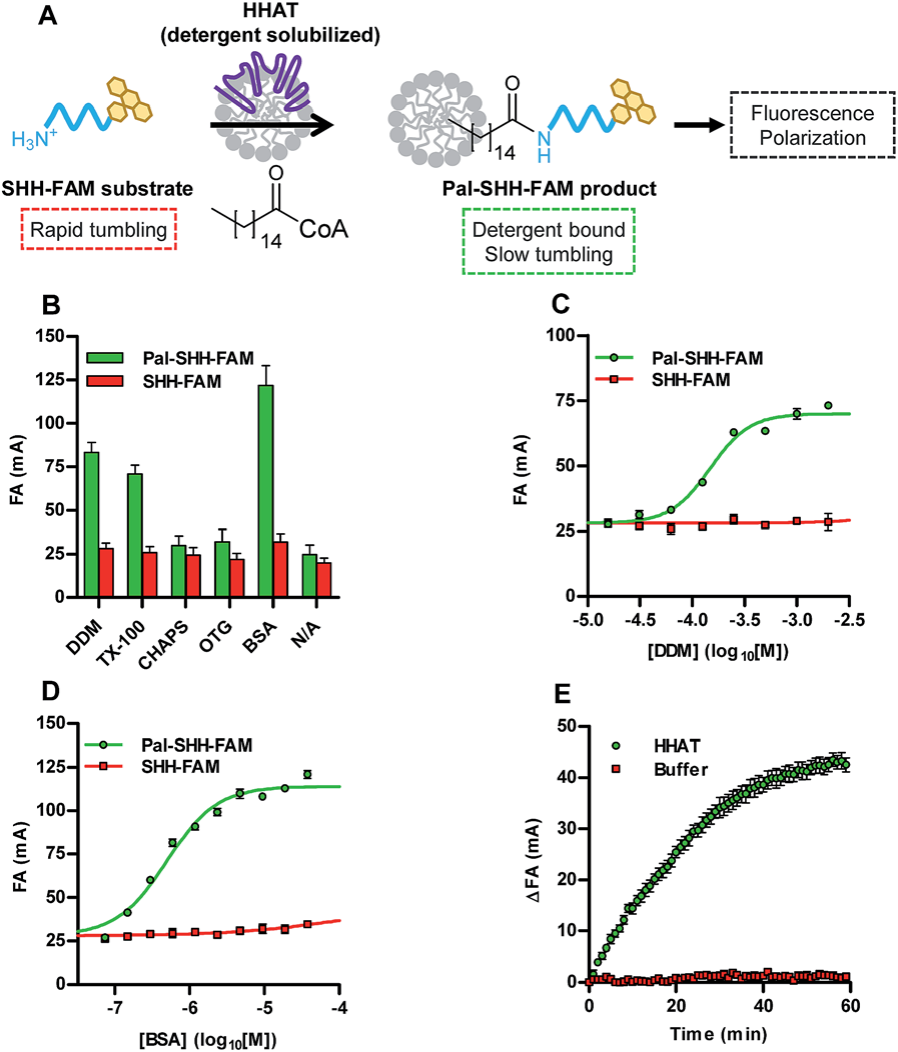
Acyl-cLIP measurement of palmitoylation of a SHH N-terminus peptide. (A) Schematic representation of the Acyl-cLIP assay for HHAT. (B) Polarisation of substrate and product SHH-FAM peptides at 1 μM and detergents at 2 mM or BSA at 0.15 mM; only BSA and detergents above their CMC increase polarised emission of the lipidated peptide. (C) DDM titration demonstrates specific Pal-SHH-FAM polarised emission above the CMC. (D) BSA titration demonstrates specific Pal-SHH-FAM polarised emission above *K*_d_. (E) Real-time analysis of SHH-FAM palmitoylation showing HHAT-dependent increase in FA over time. Data represent mean ± SEM (assays performed in duplicate, *n* = 3).

Acylation of an N-terminal peptide of Sonic Hedgehog (SHH) with palmitic (C16) acid by the enzyme hedgehog acyltransferase (HHAT) was used as a model system to validate the concept of the Acyl-cLIP format (Fig. 1A). Residues 24–33 of SHH (the N-terminus of the mature SHH signalling protein) were synthesised with fluorescein labels as substrate (SHH-FAM) and palmitoylated product (Pal-SHH-FAM) peptides (ESI, Table S1†), and prepared in mixtures with a range of detergents (2 mM) or the lipid-carrier protein bovine serum albumin (BSA, 0.15 mM). Fluorescence anisotropy (FA) measurements indicated increased polarisation for the palmitoylated peptide compared to substrate peptide in the presence of *n*-dodecyl β-d-maltoside (DDM), Triton™ X-100 (TX-100), or BSA (Fig. 1B). Detergents which did not increase Pal-SHH-FAM polarised emission had a critical micelle concentration (CMC) higher than 2 mM, strongly suggesting that detergent micelles are required for polarised emission. Titration of DDM and TX-100 with Pal-SHH-FAM demonstrated close agreement between the detergent concentration required for half-maximal polarised signal (EC_50_) and detergent CMC (DDM EC_50_ = 150 μM (95% confidence interval (CI) 130–170 μM), CMC = 170 μM; TX-100 EC_50_ = 180 μM (95% CI 140–220 μM), CMC = 200 μM; Fig. 1C and S1A†).^18^ BSA titration broadly correlated polarisation EC_50_ = 490 nM (95% CI 420–580) to the *K*_d_ of BSA for palmitic acid (104 nM) (Fig. 1D).^19^ No increase in polarised emission from non-palmitoylated SHH-FAM was observed at these concentrations. Having demonstrated a correlation between the presence of lipid-binding macromolecules/micelles and polarised emission from a palmitoylated peptide, HHAT-catalysed palmitoylation of SHH-FAM was investigated. DDM-solubilised membrane fractions from HEK293a cells stably overexpressing HHAT-FLAG-His were enriched in HHAT through Ni-NTA purification (HHAT-P100(sol)),^20^ and incubated with SHH-FAM (1 μM) and palmitoyl-CoA (Pal-CoA, 4 μM). FA measurements taken every 1 min for 1 h demonstrated increasing FA over time in the presence of HHAT-P100(sol) (Fig. 1E), with the rate of change proportional to HHAT-P100(sol) concentration (ESI, Fig. S1B†). Calculation of the total fluorescence indicated <10% quenching of SHH-FAM fluorescence during the reaction (ESI, Fig. S1C†). Unlike FA binding assays, a standard curve of palmitoylated/non-palmitoylated peptide can be used to convert observed FA values to relative concentrations (ESI, Fig. S1D†). The SHH-FAM palmitoylation reaction rate was dependent on Pal-CoA, with titration generating *V*_max_ = 0.081 pmol min^–1^ (95% CI 0.076–0.086 pmol min^–1^) and apparent *K*_M_ = 170 nM (95% CI 110–230 nM, ESI, Fig. S1E†), in good agreement with previous studies of DDM-solubilised HHAT.^13^,^20^

### Transfer of Acyl-cLIP to other lipid posttranslational modifications

**Fig. 2:**
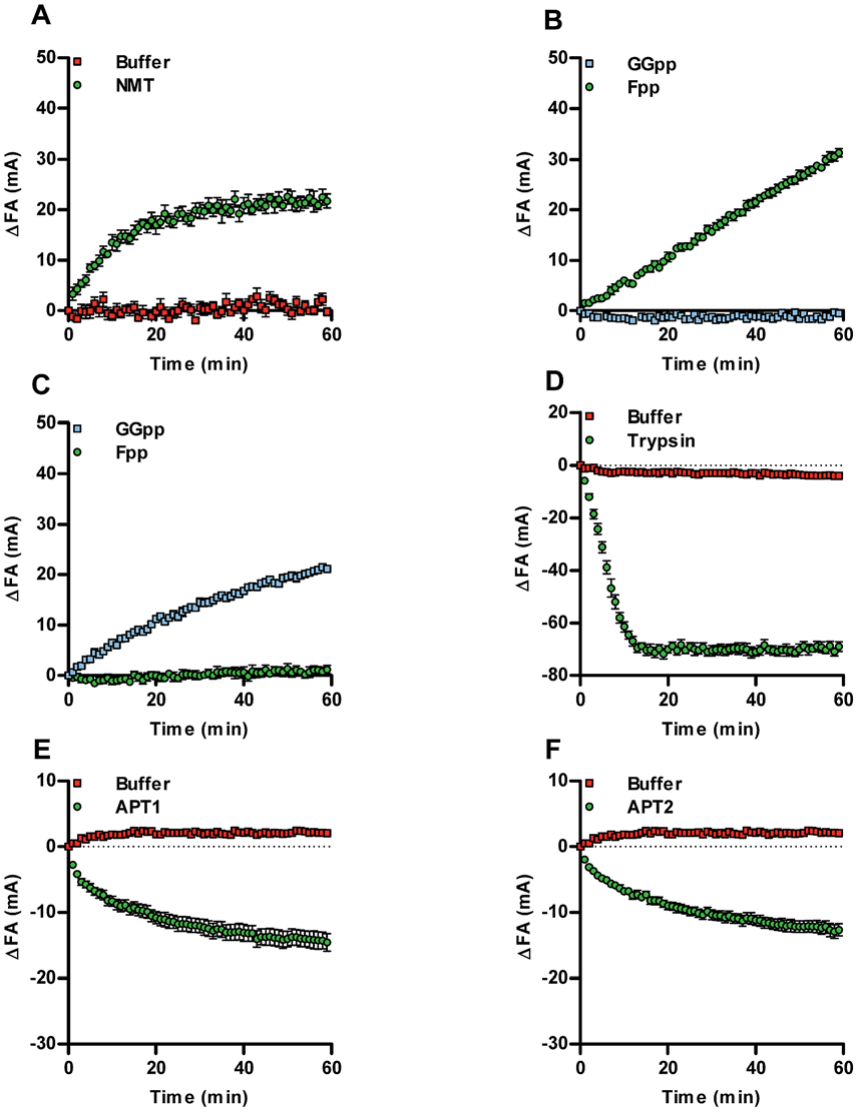
Acyl-cLIP application to lipid PTM processing enzymes. (A) NMT-mediated myristoylation of SRC-FAM. (B) FTase prenylation of FAM-KRAS CAAX-box with Fpp, but not GGpp. (C) GGTase prenylation of FAM-KRAS CAAX-box with GGpp, but not Fpp. (D) Cleavage of Pal-SHH-FAM by trypsin. (E) Deacylation of Pal-GobX-TAMRA by APT1. (F) Deacylation of Pal-GobX-TAMRA by APT2. Data represent mean ± SEM (assays performed in duplicate, *n* = 3).

The Acyl-cLIP readout is driven by the increased hydrophobicity of lipidated peptides, and therefore has the potential for universal applicability to any lipid PTM. A range of additional enzymes responsible for processing lipid PTMs were therefore tested using native lipid substrates and fluorescently labelled peptides (ESI, Table S1†). For example, *N*-myristoyl transferase (NMT) attaches myristic (C14) acid to the N-terminus of proteins, and is an attractive drug target in disease states such as cancer, malaria and the common cold.^7^,^9^,^10^ Using a SRC kinase N-terminal peptide (SRC-FAM), an NMT-dependent Acyl-cLIP signal was observed with myristoyl-CoA (Fig. 2A). Isoprenyl transferases use farnesyl pyrophosphate (Fpp) or geranylgeranyl pyrophosphate (GGpp) to lipidate CAAX-box and related motifs. Farnesyltransferase (FTase) inhibitors have been of interest to disrupt oncogenic RAS signalling; however, efficacy is limited due to compensatory prenylation by geranylgeranyl transferase type I (GGTase).^8^ Analysis of KRAS CAAX motif (FAM-KRAS) prenylation by FTase or GGTase with Fpp or GGpp demonstrated a highly reproducible increase in Prenyl-cLIP signal that occurred only with matched enzyme and lipid donors (Fig. 2B, C, and ESI, Fig. S2†). The activity of lipid transferases is complemented by hydrolytic enzymes which remove lipid PTMs, and the importance of the dynamic interplay of these processes in regulation of protein *S*-palmitoylation is increasingly appreciated.^1^,^2^,^6^ To demonstrate the feasibility of studying depalmitoylation in a Deacyl-cLIP assay, Pal-SHH-FAM was incubated with trypsin to cleave the fluorophore from the lipid moiety, which resulted in an enzyme-dependent decrease in FA signal (Fig. 2D). Acylprotein thioesterases (APTs) 1 and 2 cleave palmitate thioester bonds to cysteine residues as part of the dynamic *S*-palmitoylation cycle.^1^,^2^ Deacylation by APT1 and APT2 was therefore investigated using a TAMRA-labelled palmitoylated peptide derived from the *Legionella* effector protein GobX (Pal-GobX-TAMRA) (Fig. 2E and F). Consistent with prior assays, both APT1 and APT2 gave highly reproducible enzyme-dependent decreases in FA signal. *S*-Palmitoylation is performed by the DHHC enzyme family, of which 24 are known in human;^2^ these enzymes should also be amenable to the Acyl-cLIP assay, but generation of purified active DHHCs presents particular challenges beyond the scope of the present study.^5^

### HHAT is highly susceptible to product inhibition *in vitro*

Having demonstrated that Acyl-cLIP allowed facile access to real-time analysis of transferase and hydrolase enzymes for any type of lipid PTM, we sought to further investigate application to inhibitor discovery and validation using SHH and HHAT as a model system. The ability to generate direct, real-time measurements offers advantages in analysing lipidation kinetics; however, certain situations require stopped-assay conditions, for example during a high throughput screen (HTS). We found that addition of excess non-fluorescently labelled substrate provides a generic means to stop Acyl-cLIP assays. HHAT-catalysed palmitoylation was halted at given time points by addition of non-fluorescent SHH peptide to 20 μM (ESI, Table S1 and Fig. S3A†), which indicated a linear reaction rate over ∼30 min in agreement with real-time reaction monitoring (Fig. 1E). Stopped-signal stability was analysed over 30 min by continuous measurement, which demonstrated excellent signal stability with no signal change over time (ESI, Fig. S3B and Table S2†).

**Fig. 3:**
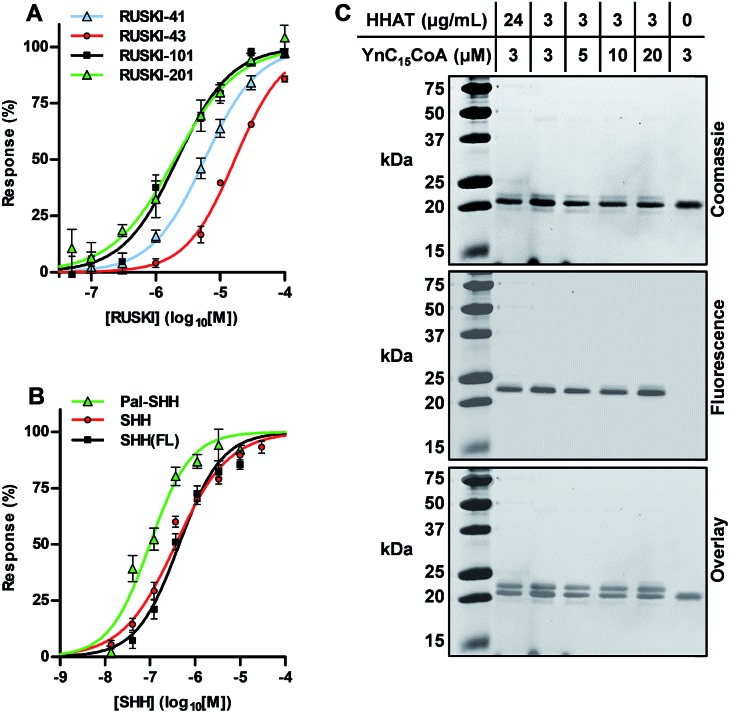
Analysis of HHAT inhibition. (A) Dose-response analysis of RUSKI compounds, demonstrating RUSKI-201 is the most potent HHAT inhibitor. (B) Dose-response analysis of SHH, SHH(FL) and Pal-SHH, indicating efficient product inhibition of HHAT. (C) SHH(FL) acylation with YnC_15_ assessed by bioorthogonal AzTB labelling and SDS-PAGE demonstrates low yield of SHH(FL) acylation. Data represent mean ± SEM (assays performed in duplicate, *n* = 3).

A class of 5-acyl-6,7-dihydrothieno[3,2,*c*]pyridines have recently been identified as low micromolar IC_50_ HHAT inhibitors in biochemical and cellular assays.^11^ Dose-response analysis of four such inhibitors over a 25 min reaction generated IC_50_ values in good agreement with prior literature (Fig. 3A, and ESI, Fig. S4 and Table S3†), demonstrating that RUSKI-201 possesses the highest potency of the RUSKI series, with RUSKI-43 having the lowest potency of compounds tested.^13^,^20^,^21^ Acyl-cLIP is equally applicable to peptide and protein competition assays; the N-terminal signalling domain of the mature full-length SHH protein (residues 24–193, SHH(FL)) was expressed and purified from *E. coli* without lipid modification. The unlabelled SHH peptide or SHH(FL) substrates were employed as competitive inhibitors of SHH-FAM palmitoylation, affording IC_50_ values of 370 nM (95% CI 300–470 nM) and 440 nM (95% CI 350–570 nM), respectively (Fig. 3B), which corresponded to approximately 50% of the SHH-FAM concentration. The very similar affinity of both the SHH N-terminus peptide and full-length SHH demonstrate that additional interactions with HHAT outside the SHH N-terminus are unlikely to play an important role in catalysis.^22^ Interestingly, the Pal-SHH peptide displayed more efficient HHAT inhibition, with an IC_50_ of 100 nM (95% CI 73–130 nM).

To cross-validate SHH(FL) acylation by HHAT and potent Pal-SHH product inhibition observed in Acyl-cLIP competition experiments, an orthogonal reporter strategy was employed. HHAT was purified to apparent homogeneity and incubated with SHH(FL) and alkyne-tagged Pal-CoA (YnC_15_-CoA), which is incorporated as the native lipid substrate.^13^ SHH(FL) acylation was detected *via* bioorthogonal ‘click chemistry’ functionalisation with azido-TAMRA-biotin (AzTB, ESI, Fig. S5†) using established copper(i)-catalysed azide–alkyne cycloaddition (CuAAC), and analysed by SDS-PAGE and in-gel fluorescence (IGF).^23^,^24^ AzTB modification causes an increase in SHH(FL) molecular weight that can be resolved by SDS-PAGE (Fig. 3C).^21^ Although only a single band was observed by either Coomassie staining or IGF, overlay showed these were separate bands, with the upper band almost undetectable by Coomassie staining. This indicated only a small proportion of SHH(FL) was acylated, and increased YnC_15_-CoA or HHAT concentrations did not increase product formation (Fig. 3C). This suggested that product inhibition may prevent complete modification of SHH(FL) in this system, in agreement with the observation from Acyl-cLIP that Pal-SHH is a highly efficient inhibitor of HHAT. During cellular SHH acylation, unloading of the Pal-SHH product may be performed by as yet unidentified chaperone proteins, or result from partition of the Pal-SHH product into the ER membrane.

### Acyl-cLIP displays excellent characteristics for high-throughput screening

**Fig. 4:**
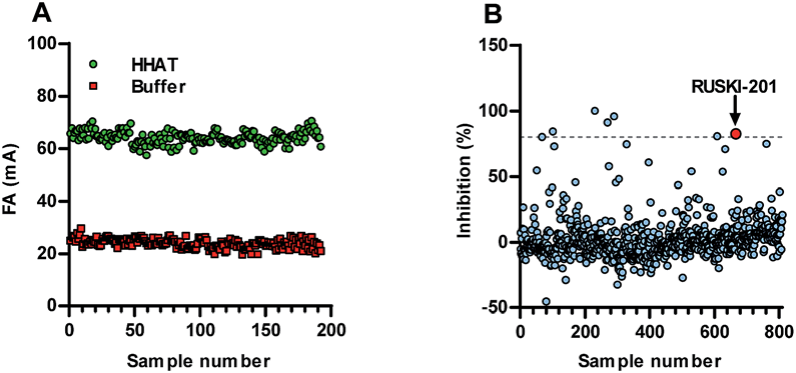
Suitability of Acyl-cLIP for HTS campaigns. (A) Stopped signal from full 384-well plate of positive (HHAT) and negative (buffer) controls indicating excellent HTS suitability (*Z*′ = 0.69). (B) Screening of 775 FDA-approved drugs for inhibition of HHat, including RUSKI-201 as blind positive control. Hits defined as giving >80% inhibition at 25 μM.

Acyl-cLIP provided accurate analysis of peptide, protein and small-molecule inhibitors, therefore its application in an HTS-compatible format to identify new inhibitors was investigated. Implication of Hedgehog (HH) signalling in the formation and maintenance of cancers has driven interest in the therapeutic potential of small-molecule HH-pathway inhibitors.^25^ Indeed, inhibitors of the HH pathway component Smoothened have reached the clinic, although their efficacy is compromised by the rapid emergence of resistance mutations that block inhibitor binding.^26^,^27^ HHAT inhibition offers a new route to arrest HH signalling, and the likelihood of developing a clinically applicable HHAT inhibitor would be greatly increased by identification of novel chemical series. The *Z*-factor (*Z*′) of an assay is a measure of signal window relative to signal noise, with *Z*′ > 0.5 indicating an excellent assay for use in an HTS.^28^ Real-time reaction monitoring of a full 384-well plate allowed selection of an HHAT concentration and reaction time delivering *Z*′ = 0.51 under linear reaction progression at 1.7 h (ESI, Fig. S6†). Established stopped-assay conditions were then employed after a 1.5 h reaction to afford *Z*′ = 0.69 from 384 wells (Fig. 4A). Real-time monitoring of the stopped signal indicated excellent signal stability with *Z*′ > 0.5 for 5 h (ESI, Fig. S7†).

To demonstrate compatibility with HTS, a pilot Acyl-cLIP screen was conducted using a library of 775 FDA-approved drug molecules, alongside the most potent small-molecule HHAT inhibitor, RUSKI-201, as a blind positive control. Compounds were screened at 25 μM and six hit molecules were identified showing >80% inhibition (0.8% hit rate), in addition to RUSKI-201 (Fig. 4B, and ESI, Fig. S8†). Counter-screening using in-cell labelling of SHH with YnC_15_ and click-chemistry functionalisation identified two hits, bromocriptine and clomipramine, alongside RUSKI-201 blind controls which showed inhibition of SHH acylation by IGF. However, bromocriptine and clomipramine did not show inhibition of SHH acylation when assessed for increased molecular weight by anti-SHH blotting (ESI, Fig. S9†). These two new hits were therefore further triaged alongside RUSKI-201 in a SHH-Light2 cellular signalling assay, which expresses *Firefly* luciferase under control of a SHH-inducible promoter, alongside a constitutive *Renilla* luciferase control for cellular viability.^29^ Bromocriptine displayed general cytotoxicity, whereas clomipramine only inhibited HH signalling at >30 μM, which was most likely due to non-specific effects as reflected in decreased viability at high concentrations in MTS assays (ESI, Fig. S10†).

## Conclusions

Lipid transferases and hydrolases are emerging as attractive and tractable therapeutic targets in a number of disease states; however, drug discovery efforts are hindered by challenges in high-throughput biochemical assays. We report here a new method for monitoring the activity of lipid transferase and hydrolase enzymes, which allows highly accurate real-time and stopped-assay measurement of lipidation. Acyl-cLIP uses an intrinsic property of lipid modification, hydrophobicity, to drive the assay readout, therefore offering advantages over existing assay formats, including enhanced versatility, safety, economy and increased throughput. Furthermore, the method can be readily applied to a range of targets and lipid PTMs thanks to the use of native lipid substrates and synthetically-accessible fluorescent peptides. Bioorthogonal lipid probes, for example incorporating alkyne tags, are equally compatible with the presented assay format. Indeed, we recently reported novel alkyne-farnesyl and -geranylgeranyl probes for global profiling of protein prenylation in live cells, and demonstrated in Prenyl-cLIP assays that the probes reproduce the enzyme and CAAX-box selectivity of Fpp and GGpp.^8^

Acyl-cLIP was found to be universally applicable to enzymes involved in processing lipid PTMs. In developing these assays the design of substrate peptides was found to be a critical consideration. Lipophilic or lengthy peptide substrates can result in a decrease or loss of signal window between substrate and product. In developing an Acyl-cLIP assay for a new target both substrate and product peptides should be analysed to identify conditions that afford a maximal assay window. As an enzymatic assay, Acyl-cLIP formats require substrate concentrations defined by the target enzyme *K*_m_, in contrast to FA binding assays where nM concentrations of fluorescent peptides are typically used. It is therefore important that spectrophotometer settings are adjusted to avoid saturation of the detector. Use of initial velocity conditions (<10% substrate consumption) in enzyme assays means that the magnitude of signal window is smaller than for FA binding assays; however, real-time signal monitoring as presented here allows highly accurate determination of enzyme activity under these conditions.

Investigation of *N*-palmitoylation by HHAT provided insights into both substrate recognition and product inhibition, as well as demonstrating that Acyl-cLIP possesses excellent characteristics to enable future HTS campaigns. A pilot screen of 775 FDA approved drugs was conducted; although no new HHAT inhibitors were identified, the blind positive control compound RUSKI-201 was successfully identified. We note that the potential teratogenic effect of HH pathway inhibition diminishes the likelihood of FDA-approved drugs possessing off-target HHAT inhibition; however, this proof of principle study demonstrated the suitability of Acyl-cLIP and counter-screening assays to identify and triage HHAT inhibitors. We have subsequently successfully completed a full HTS to identify new HHAT inhibitors, the results of which will be disclosed in due course.

In summary, we present Acyl-cLIP as a versatile and HTS-compatible biochemical assay for lipid PTMs, which overcomes the limitations of existing methods. As enzymes responsible for lipid PTM processing continue to emerge as an increasingly tractable and promising classes of drug targets, we anticipate that this new assay format will greatly expedite future studies and medicinal chemistry programs.

## Conflicts of interest

There are no conflicts to declare.

## Supplementary Material

Supplementary information
