# Supplementary material for: Acylation-coupled lipophilic induction of polarisation (Acyl-cLIP): a universal assay for lipid transferase and hydrolase enzymes
Source: Chem Sci. 2019 Jul 1;10(39):8995–9000. doi: 10.1039/c9sc01785b (PMC6855259; doi:10.1039/c9sc01785b)
Supplement: Supplementary file 1 [file SC-010-C9SC01785B-s001.pdf]

Electronic Supplementary Material (ESI) for Chemical Science.
This journal is © The Royal Society of Chemistry 2019

Supporting Information

# Acylation-coupled Lipophilic Induction of Polarisation (Acyl-cLIP): a Universal Assay for Lipid Transferase and Hydrolase Enzymes

Thomas Lanyon-Hogg,*[a] Markus Ritzefeld,[a] Lea Sefer,[b] Jasmine K. Bickel,[a] Amalie F. Rudolf,[b] Nattawadee Panyain,[a] Ganka Bineva-Todd,[c] Corey A. Ocasio,[c] Nicola O'Reilly,[c] Christian Siebold,[b] Anthony I. Magee,[d] and Edward W. Tate*[a]

[a] Department of Chemistry, Imperial College London, London, W12 0BZ, UK.
E-mail: t.lanyon-hogg@imperial.ac.uk; e.tate@imperial.ac.uk; Tel: (+44) 0207 5943752; (+44) 0207 5945821.

[b] Division of Structural Biology, Wellcome Centre for Human Genetics, University of Oxford, Oxford, OX3 7BN, UK.

[c] The Francis Crick Institute, London NW1 1AT, UK.

[d] Molecular Medicine Section, National Heart & Lung Institute, Imperial College London, London, SW7 2AZ, UK

## Table of Contents


Supplementary Results ........ 3

Supplementary Figures ........ 4

Supplementary Tables ........ 15

Materials and Methods ........ 18

- Abbreviations ........ 18
- Chemical Synthesis ........ 18
  - General Information ........ 18
  - General Procedure for Solid Phase Peptide Synthesis ........ 18
  - Pal-SHH ........ 19
  - SRC-FAM ........ 19
  - FAM-KRAS ........ 19
  - Pal-GobX-TAMRA ........ 20
- Biological and Biochemical Methods ........ 21
  - General Information ........ 21
  - Recombinant Protein Production ........ 22
  - HHAT-P100(sol) ........ 22
  - Purified HHAT ........ 22
  - Purified SHH(FL) ........ 22
  - APT1 and APT2 ........ 23
  - Fluorescence Anisotropy ........ 23
  - HHAT Acyl-cLIP Assay ........ 24
  - NMT Acyl-cLIP Assay ........ 24
  - FTase/GGTase Prenyl-cLIP Assay ........ 24
  - Trypsin Deacyl-cLIP Assay ........ 24
  - APT1/2 Deacyl-cLIP Assay ........ 25
  - SHH-Light2 Signalling Assay ........ 25
  - In-Cell YnC$_{15}$ Tagging Assay ........ 25
  - MTS Assay ........ 26

References ........ 27

**Supplementary Results**

Supplementary Figure S1. Analysis of SHH palmitoylation by Acyl-cLIP. ..............................4

Supplementary Figure S2. Prenyl-cLIP analysis of KRAS lipidation.......................................5

Supplementary Figure S3. Acyl-cLIP analysis of stopped-assay conditions. ..........................6

Supplementary Figure S4. Small-molecule inhibitors of HHAT. .............................................7

Supplementary Figure S5. Structure of azido-TAMRA-PEG-biotin (AzTB) ‘capture’ reagent..8

Supplementary Figure S6. HTS suitability of real-time Acyl-cLIP assay.................................9

Supplementary Figure S7. HTS suitability of stopped Acyl-cLIP assay. ................................10

Supplementary Figure S8. Pilot screen hit identity. ............................................................11

Supplementary Figure S9. Inhibition of SHH acylation in cells by pilot screen hits. ..............12

Supplementary Figure S10. Pilot screen hits effects on HH signalling and cellular viability. .13

Supplementary Figure S11. Recombinant protein production. .............................................14

Table S1. Peptide sequences. ..........................................................................................15

Table S2. Analysis of stopped signal stability. ....................................................................16

Table S3. $IC_{50}$ values from dose-response analysis of HHAT inhibition. ..............................17

## Supplementary Figures

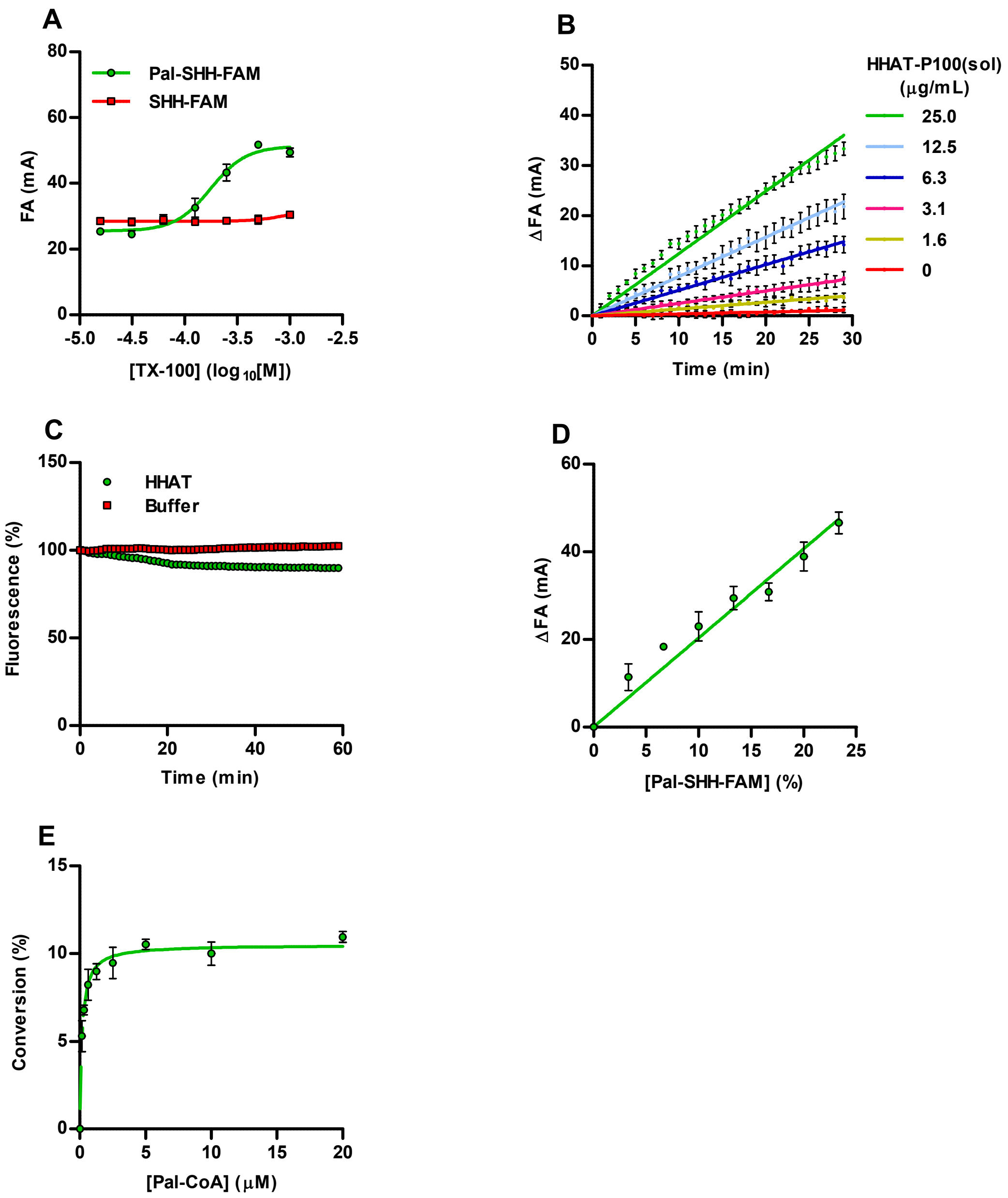


**Supplementary Figure S1. Analysis of SHH palmitoylation by Acyl-cLIP**.

(A) Triton™ X-100 (TX-100) titration in the presence of Pal-SHH-FAM and SHH-FAM affording a half-maximal response ($EC_{50}$) = 180 µM (95% confidence interval (CI) 140-230 µM) for Pal-SHH-FAM polarisation, in good agreement with the detergent critical micelle concentration (CMC, 200 µM).[1] (B) Serial dilution of HHAT-P100(sol) demonstrating Acyl-cLIP signal is proportional to enzyme concentration. (C) Total fluorescence intensity values for palmitoylation reaction shown in Figure 2E, showing <10% fluorescence quenching from lipidation. (D) Standard curve of mixed palmitoylated and non-palmitoylated SHH-FAM at 1 µM total concentration. (E) Pal-CoA concentration-dependent Acyl-cLIP signal under initial velocity conditions gives $V_{max}$ = 0.081 pmol/min (95% CI 0.076-0.086 pmol/min) and apparent $K_M$ = 170 nM (95% CI 110-230 nM). Data represent mean ± SEM (assays performed in duplicate, n = 3).

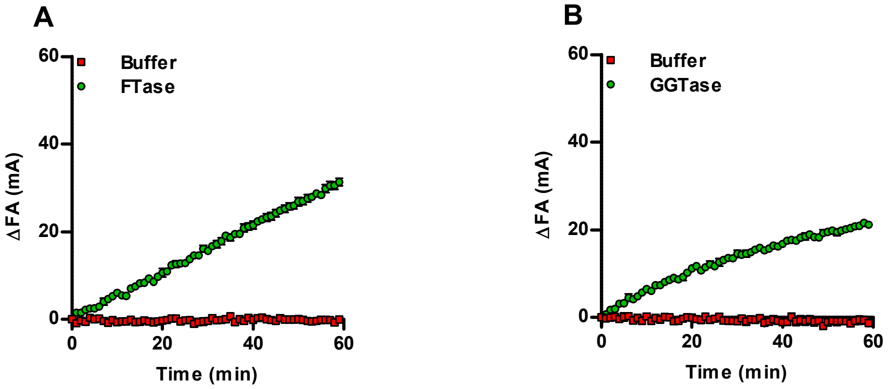


**Supplementary Figure S2. Prenyl-cLIP analysis of KRAS lipidation.**

Controls corresponding to Figure 2B and 2C. (A) Reaction of farnesyl pyrophosphate and FAM-KRAS in the presence or absence of FTase. (B) Reaction of geranylgeranyl pyrophosphate and FAM-KRAS in the presence or absence of GGTase. Data represent mean ± SEM (assays performed in duplicate, n = 3).

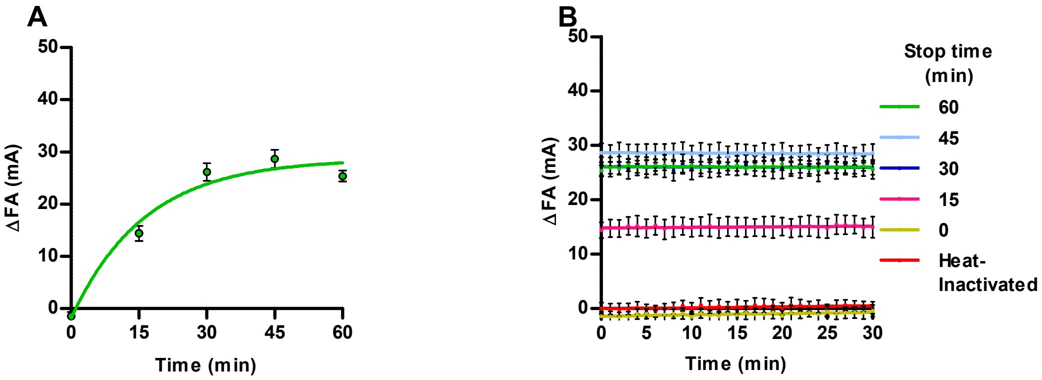


**Supplementary Figure S3. Acyl-cLIP analysis of stopped-assay conditions.**

(A) HHAT-P100(sol)-catalysed SHH-FAM palmitoylation halted at indicated time points by addition of SHH to 20 μM, demonstrating reaction progression in agreement with real-time measurements. Signal background corrected against heat-inactivated HHAT-P100(sol). (B) Stopped-assay signal stability analysed through real-time monitoring of samples shown in Figure S3A, demonstrating no statistically significant change in signal with time (Table S2). Data represent mean ± SEM (assays performed in duplicate, n = 3).

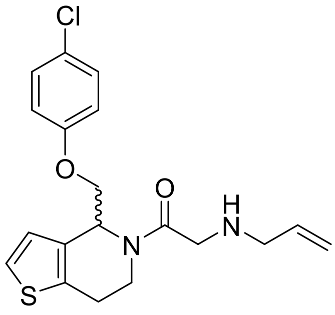

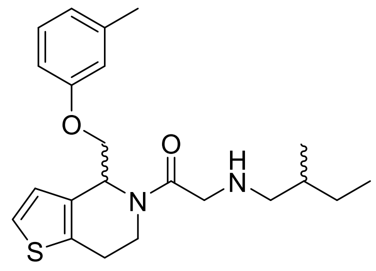

RUSKI-41 RUSKI-43

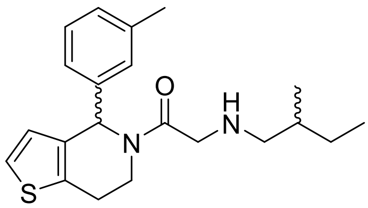

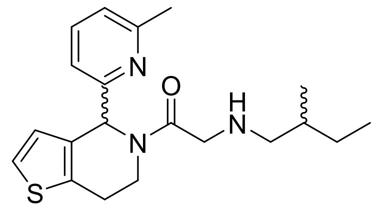

RUSKI-101 RUSKI-201

**Supplementary Figure S4. Small-molecule inhibitors of HHAT.**

Structures of RUSKI small-molecule inhibitors of HHAT. Dose-response analysis of RUSKI inhibitors is shown in Figure 3A, and half-maximal inhibition values ($IC_{50}$) with 95% CI for all inhibitors given in Table S3.

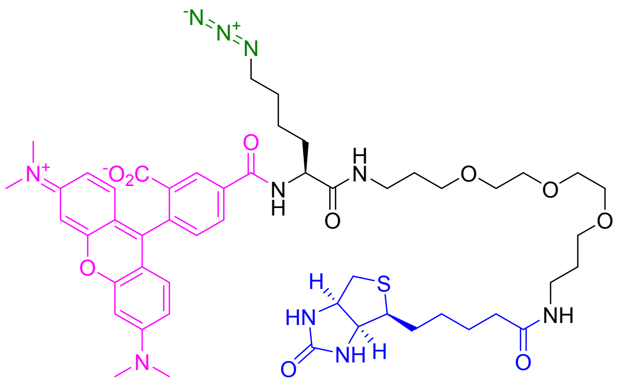

**Supplementary Figure S5. Structure of azido-TAMRA-PEG-biotin (AzTB) 'capture' reagent.**

AzTB contains three functional moieties; an azide (green) for bioorthogonal 'click chemistry' ligation to alkyne-containing molecules using a copper(I)-catalysed azide-alkyne cycloaddition reaction; a TAMRA fluorophore (pink) for in-gel fluorescence analysis; a biotin group (blue) for streptavidin-based enrichment or detection.[2]

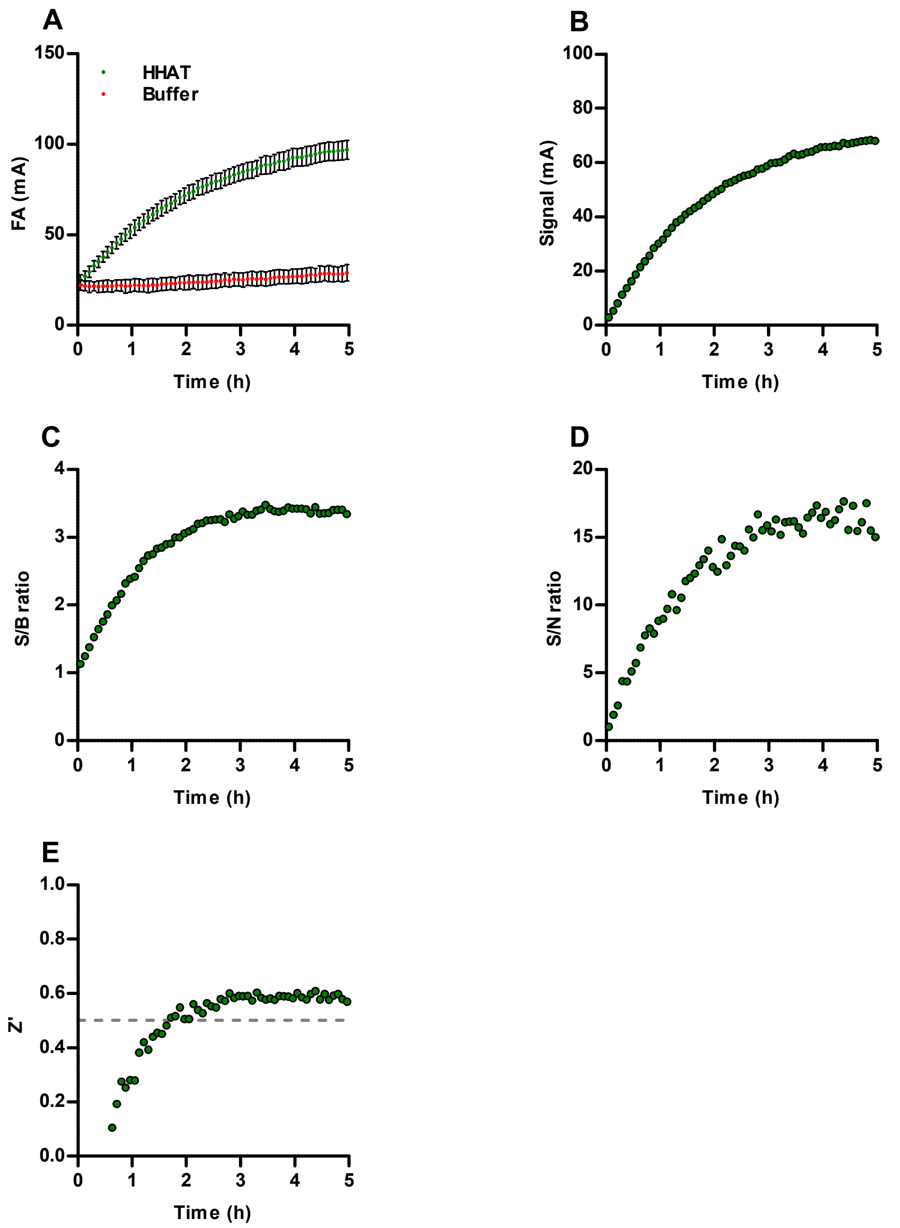


**Supplementary Figure S6. HTS suitability of real-time Acyl-cLIP assay.**

Reaction performed on 382 samples (192 positive- and 190 negative-control samples) and monitored using real-time measurement. (A) Raw fluorescence anisotropy readings for samples containing HHAT or DDM solubilisation buffer. Data represent mean ± SD. (B) Signal window development with time indicating linear reaction progression up to ~1.7 h. (C) Signal-to-background (S/B) ratio increase with reaction progression. (D) Signal-to-noise (S/N) ratio increase with reaction progression. (E) Z-factor (Z') increase with reaction progression. At t = 1.7 h the assay generates Z' = 0.51, indicating an excellent assay format.

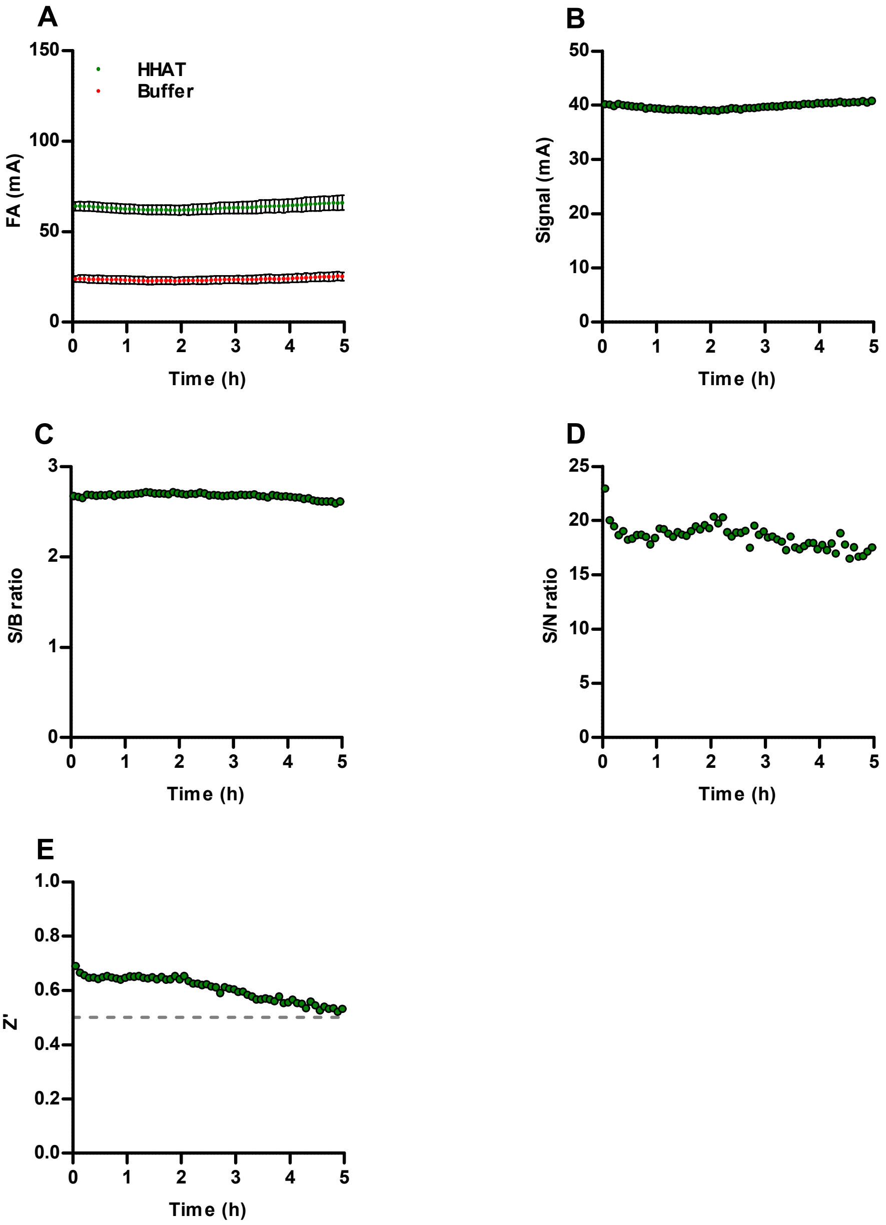


**Supplementary Figure S7. HTS suitability of stopped Acyl-cLIP assay.**

Full 384-well plate (192 positive- and 192 negative-control samples) stopped after 1.5 h reaction (20 µM SHH) and monitored using real-time measurement. (A) Raw fluorescence anisotropy readings for samples containing HHAT or DDM solubilisation buffer, indicating high signal stability. Data represent mean ± SD. (B) Signal window, demonstrating a highly stable signal with time. (C) S/B ratio stability, with $S/B_{max}$ = 2.7. (D) S/N ratio stability, with $S/N_{max}$ = 23. (E) Z' stability with time, demonstrating Z' > 0.5 for 5 h with $Z'_{max}$ = 0.69.

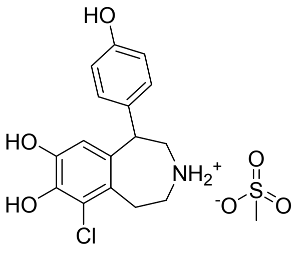

Fenoldopam Mesylate

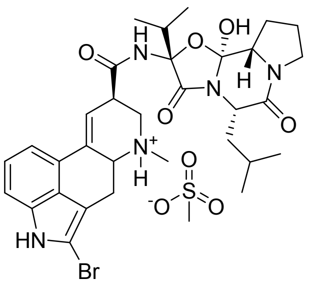

Bromocriptine Mesylate

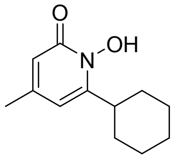

Ciclopirox

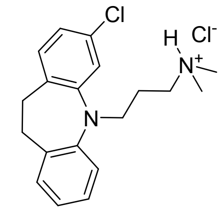

Clomipramine·HCl

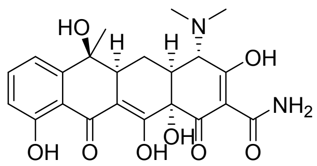

Tetracycline

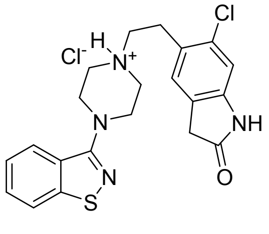

Ziprasidone·HCl

**Supplementary Figure S8. Pilot screen hit identity.**

A library of 775 FDA-approved molecules were screened at 25 µM for HHAT inhibition using the Acyl-cLIP assay, with hits defined as compounds showing >80% inhibition of HHAT activity.

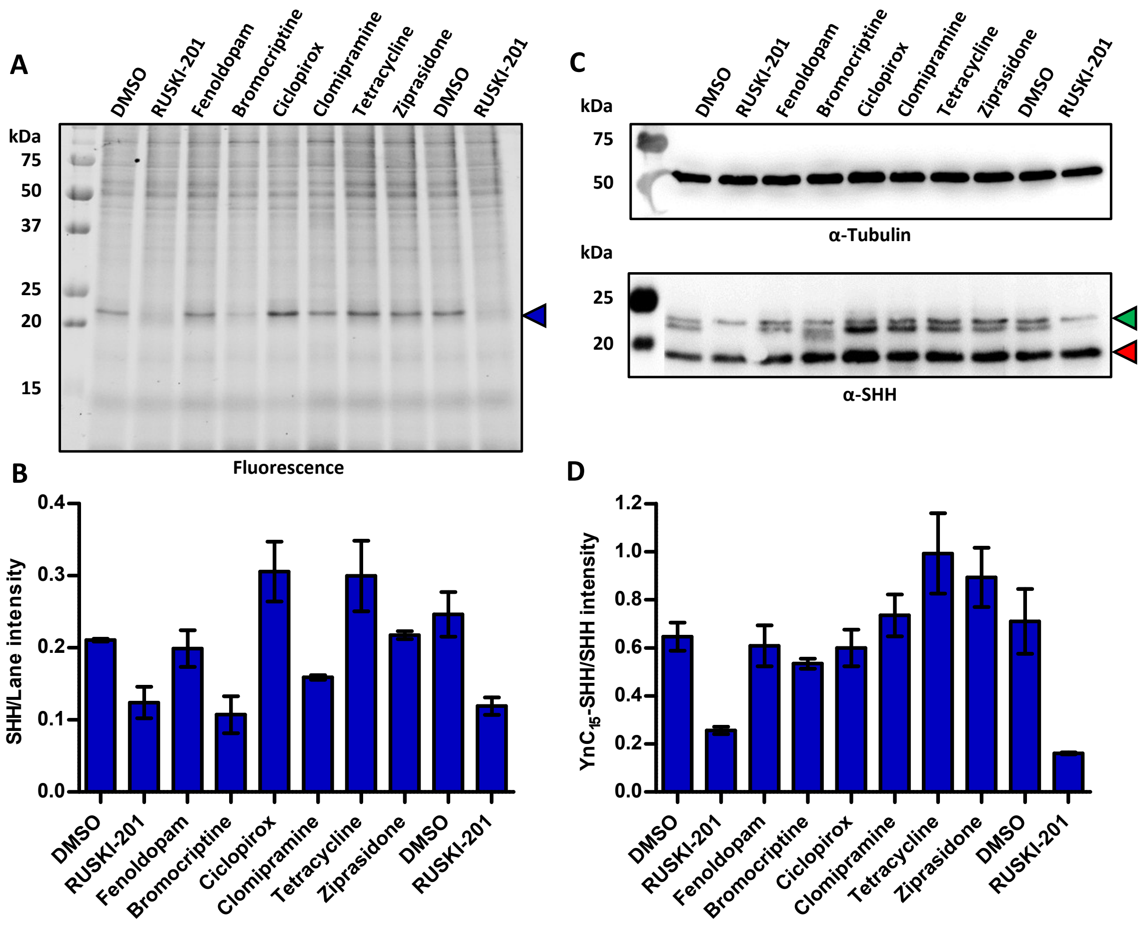


**Supplementary Figure S9. Inhibition of SHH acylation in cells by pilot screen hits**.

HEK-293 cells stably overexpressing SHH (HEK-293 SHH$^+$) were treated with hit compounds or DMSO vehicle and $YnC_{15}$, followed by lysis and ligation to AzTB. Four blind controls were included; two RUSKI-201 positive controls and two DMSO negative controls. (A) In-gel fluorescence analysis of in-cell $YnC_{15}$ tagging. Image representative of two separate experiments. $YnC_{15}$-SHH band quantified indicated by blue arrow. (B) Quantification of in-gel fluorescence through densitometry, displayed as the ratio SHH band intensity to total lane intensity. Bromocriptine, Clomipramine and RUSKI-201 all display inhibition of SHH $YnC_{15}$-tagging. Data represent mean ± SEM (n = 2). (C) Western blot analysis of in-cell $YnC_{15}$ tagging using α-Tubulin (loading control) and α-SHH. $YnC_{15}$-tagging and click functionalisation results in an increase in SHH molecular weight observable by α-SHH blotting, indicated by a green arrow. Untagged SHH indicated by a red arrow. Image representative of two separate experiments. (D) Quantification of α-SHH blotting by densitometry, displayed as the ratio of $YnC_{15}$-tagged SHH to untagged SHH. Only RUSKI-201 displayed inhibition of SHH $YnC_{15}$ tagging. Data represent mean ± SEM (n = 2).

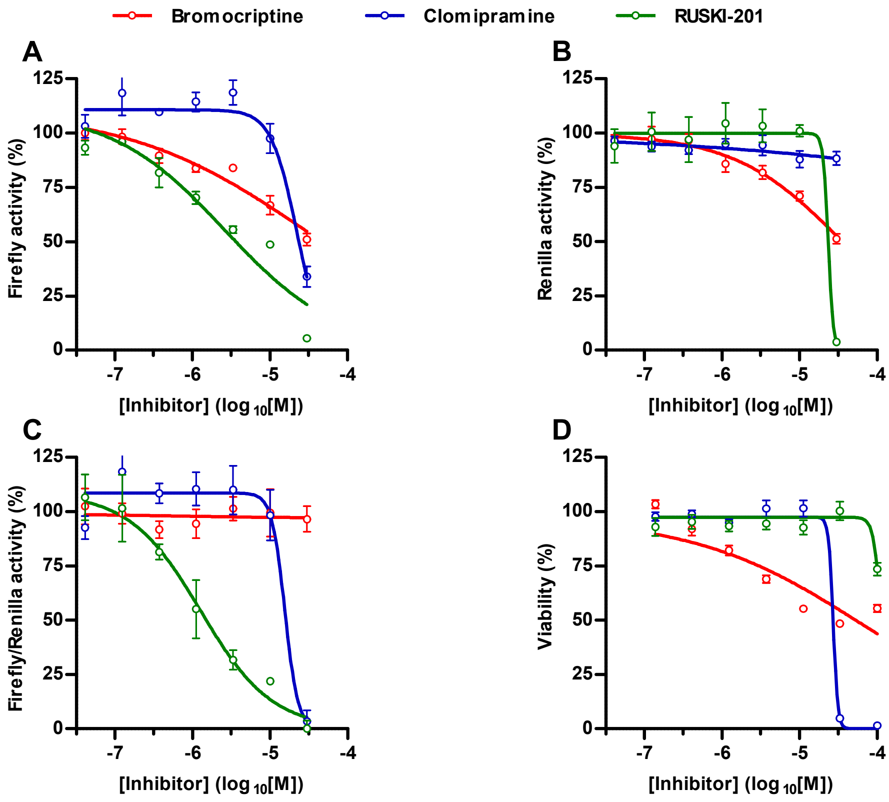


**Supplementary Figure S10. Pilot screen hits effects on HH signalling and cellular viability.**

The effects of Bromocriptine (red), Clomipramine (blue) and RUSKI-201 (green) on HH signalling and viability were analysed using the Light2 signalling assay. (A) HH-dependent *Firefly* luciferase activity in Light2 cells in response to compound treatment. (B) Constitutive *Renilla* luciferase activity in Light2 cells in response to compound treatment. (C) Combined *Firefly*/*Renilla* activity, demonstrating that only RUSKI-201 exhibits an on-target window where HHAT is inhibited without cytotoxic effects. (D) MTS viability assay for effect of compounds on HEK-293 SHH$^+$ cells, demonstrating that although Clomipramine decreases HH signalling at 30 μM, this effect is most likely do to non-specific effects as demonstrated by the cytotoxicity at this concentration to HEK-293 SHH$^+$ cells. Data represent mean ± SEM (n = 3).

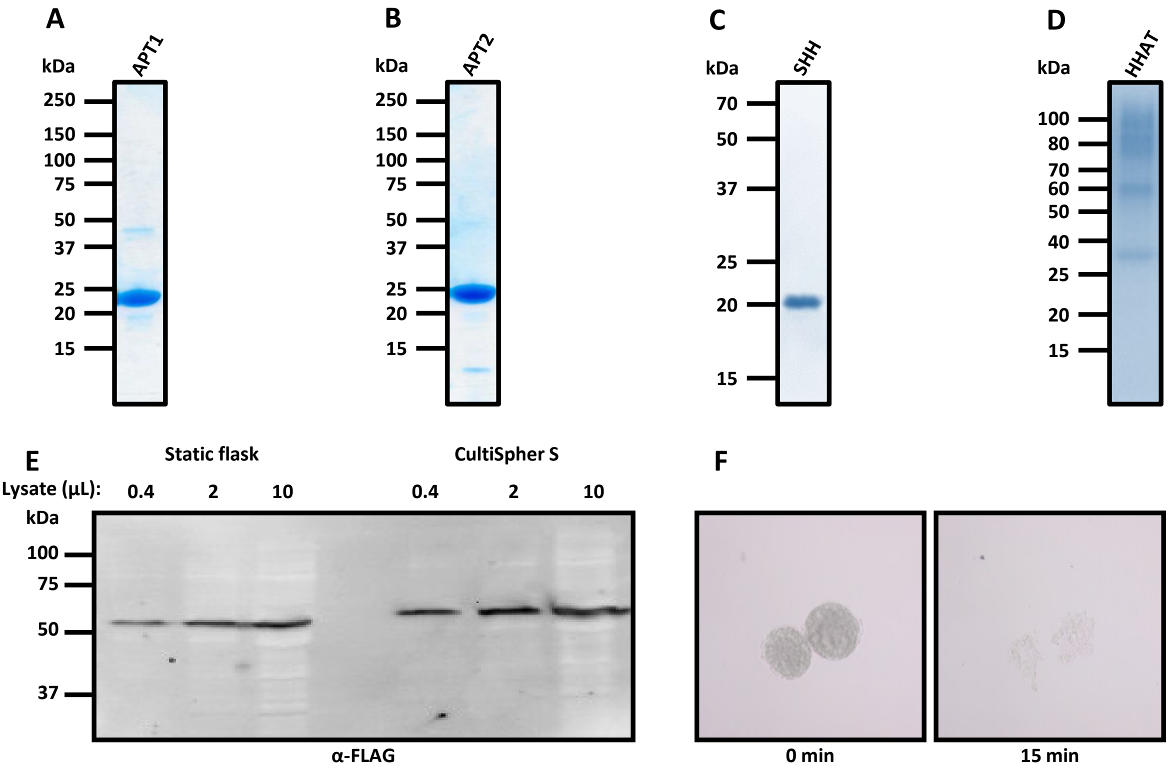


**Supplementary Figure S11. Recombinant protein production.**

Recombinant proteins were produced and purified as described in Materials and Methods for use in Acyl-cLIP assays. (A) Coomassie stain of purified APT1. (B) Coomassie stain of purified APT2. (C) Coomassie stain of purified SHH. (D) Coomassie stain of purified HHAT. As a multipass transmembrane protein, HHAT cannot be boiled prior to SDS-PAGE and can be observed to migrate as dimeric or higher oligomeric species.[3] (E) α-FLAG immunoblot showing that HHAT-FLAG-His is expressed by growth in either static flasks or in stirred CultiSpher S microcarrier culture. α-FLAG-HRP immunoblotting was performed as previously described.[4] (F) Visual monitoring of CultiSpher S digestion by trypsinisation, showing microcarrier digestion within 15 min treatment.

## Supplementary Tables

| Peptide | Residues | Sequence |
|---|---|---|
| SHH | 24-33* | CGPGRGFGKRK-$CONH_2$ |
| Pal-SHH | 24-33* | Pal-CGPGRGFGKRK-$CONH_2$ |
| SHH-FAM | 24-33* | CGPGRGFGKR(K-FAM)G-$CONH_2$ |
| Pal-SHH-FAM | 24-33* | Pal-CGPGRGFGKR(K-FAM)G-$CONH_2$ |
| SRC-FAM | 2-9* | GSNKSKPK(K-FAM)G-$CONH_2$ |
| FAM-KRAS | 184-189 | FAM-βAKKCIIM-$CO_2H$ |
| Pal-GobX-TAMRA | 171-190 | Ac-QSRS(C-Pal)WSLFLYSIFGGKDNR-NH-$C_2H_4$-NH-TAMRA |

**Table S1. Peptide sequences**.
Amino acid sequences of fluorescently-labelled and unlabelled peptides synthesised as described in Materials and Methods. Peptides were used as described in validation and enzymatic assays. Residue numbers donated * indicate peptides that are the N-terminus of the mature protein after proteolytic processing.

| Time (min) | 0 | 15 | 30 | 45 | 60 | Heat Inactivated |
|---|---|---|---|---|---|---|
| Gradient (mA/min) | 0.023 ± 0.019 | 0.010 ± 0.033 | -0.002 ± 0.033 | -0.009 ± 0.030 | -0.002 ± 0.020 | 0.018 ± 0.020 |
| Deviation from zero: | | | | | | |
| P value | 0.211 | 0.760 | 0.943 | 0.770 | 0.937 | 0.385 |
| Significance | Not Significant | Not Significant | Not Significant | Not Significant | Not Significant | Not Significant |

**Table S2. Analysis of stopped signal stability.**

HHAT-P100(sol)-catalysed SHH-FAM palmitoylation was halted at indicated time points by addition of SHH to 20 μM, and FA readings taken continuously for 30 min to determine stopped-assay signal stability (Supplementary Figure S3B). A significant P value was taken to be <0.05.

| Inhibitor | $IC_{50}$ (μM) | 95% CI (μM) |
|---|---|---|
| RUSKI-41 | 5.7 | 4.5-7.3 |
| RUSKI-43 | 17 | 14-21 |
| RUSKI-101 | 2.1 | 1.7-2.7 |
| RUSKI-201 | 2.0 | 1.4-2.8 |
| SHH(FL) | 0.44 | 0.35-0.57 |
| SHH | 0.37 | 0.30-0.47 |
| Pal-SHH | 0.10 | 0.073-0.13 |

**Table S3. $IC_{50}$ values from dose-response analysis of HHAT inhibition**.

Values determined from Acyl-cLIP analysis of HHAT-P100(sol) activity. Dose-response curves are shown in Figure 3A and small molecule inhibitor structures shown in Supplementary Figure S4. $IC_{50}$ values and 95% CI range were extracted by nonlinear regression using a four parameter sigmoidal dose response model (assays performed in duplicate, n = 3).

## Materials and Methods

### Abbreviations

DCM (dichloromethane); DIPEA (*N*,*N*-diisopropylethylamine); DMF (dimethylformamide); DTT (dithiothreitol); EDTA (ethylenediaminetetraacetic acid); FA (formic acid); FAM (5/6-carboxyfluorescein); HATU (1-[bis(dimethylamino)methylene]-1*H*-1,2,3-triazolo[4,5-b]pyridinium 3-oxid hexafluorophosphate); HBTU (*N*,*N*,*N*′ ,*N*′ -tetramethyl-*O*-(1*H*-benzotriazol-1-yl)uronium hexafluorophosphate); HCTU (1-[bis(dimethylamino)methylen]-5-chlorobenzotriazolium 3-oxide hexafluorophosphate); HEPES (4-(2-hydroxyethyl)-1-piperazineethanesulfonic acid); HOBt (hydroxybenzotriazole); IPTG (isopropyl β-D-1-thiogalactopyranoside); ivDde (1-(4,4 dimethyl2,6-dioxocyclohexylidene)-3-methylbutyl); MMT (monomethoxytrityl); MTS (3-(4,5-dimethylthiazol-2-yl)-5-(3-carboxymethoxyphenyl)-2-(4-sulfophenyl)-2H-tetrazolium); NMM (*N*-methylmorpholine); NMP (*N*-methyl-2-pyrrolidone); PBS (phosphate buffered saline); PBS-T (phosphate buffered saline plus Tween-20 (0.1% (v/v))); RT (room temperature); TBTA (tris[(1-benzyl-1H-1,2,3-triazol-4-yl)methyl]amine); TCEP (tris(2-carboxyethyl)phosphine hydrochloride);TFA (trifluoroacetic acid); TFE (trifluoroethanol); TIS (triisopropylsilane); TMP (trimethoxyphenylthio); TRIS (tris(hydroxymethyl)aminomethane).

### Chemical Synthesis

### General Information

All reagents were purchased from commercial sources (Sigma-Aldrich, Fisher Scientific, Acros Organics, Novabiochem) and used without further purification.

SHH, SHH-FAM and Pal-SHH-FAM were synthesised as previously described.[4] RUSKI inhibitors were synthesised as previously described.[5] AzTB was synthesised as previously described.[2]

Mixtures described as % represent v/v unless otherwise stated.

### General Procedure for Solid Phase Peptide Synthesis

Peptides were synthesised on an Intavis ResPep SL Automated Peptide Synthesizer (Intavis Bioanalytical Instruments, Germany) on Rink amide resin using the *N*(α)-Fmoc/$^t$Bu solid phase orthogonal protection protocol, unless otherwise stated. Resins were swelled in DMF prior to synthesis. Synthetic cycles consisted of N-terminal Fmoc deprotection using piperidine (20%) in DMF for 30 min, followed by washing with DMF. *N*(α)-Fmoc protected amino acids (0.6 M, 4.3 eq) in NMP were activated by mixing with NMM (4 M, 10 eq) in NMP, and HBTU (0.6 M, 4.3 eq) before addition to the resin. Upon completion, the resin was washed with DCM and the coupling repeated. After coupling, the resin was washed with $Ac_2O$ (5%) in DMF to cap unreacted amines before washing with DMF. The deprotection/double-coupling/capping cycle was repeated for each amino acid followed by Fmoc deprotection of the final residue.

Peptides were cleaved from the resin using appropriate cleavage cocktails, as described. The resultant peptide solution was concentrated under a stream of nitrogen and crude peptide

precipitated with cold $Et_2O$ (15 mL). The precipitated peptide was collected by centrifugation (4000 rpm, 5 min, 4 °C) and solvents removed by decanting. The peptide pellet was re-dissolved for purification by reverse phase HPLC.

**Pal-SHH**

The peptide was synthesised following the general procedure. Palmitic acid (3 eq) in DMF was activated with HATU (0.6 M, 4.3 eq) in DMF.

The peptide was cleaved from the resin using Reagent K cleavage cocktail (5 mL, 82.5% TFA, 5% phenol, 5% $H_2O$, 5% thioanisole, 2.5% EDT) for 2 h at RT with agitation and isolated as described. The peptide pellet was re-dissolved in MeOH:$H_2O$ (20:80) supplemented with FA (0.1%) and purified by preparative HPLC-MS using a linear solvent gradient of 20-98% MeOH (0.1% FA) in $H_2O$ (0.1% FA) over 18 min at a flow rate of 20 mL/min. Collected fractions were pooled and lyophilised to afford the required peptide as a white solid.

MS (TOF MS ES+), m/z: calculated for $C_{65}H_{114}N_{20}O_{12}S$ $[M+H]^+$: 1400.81, found: 1400.19; Analytical RP-HPLC: Gradient: 5-98% MeOH (0.1% FA) in $H_2O$ (0.1% FA) over 18 min, RT = 11.86 min.

**SRC-FAM**

The peptide was synthesised following the general procedure. FAM was incorporated using *N*(α)-Fmoc-Lys(5/6-FAM)-OH (1.2 eq) in DMF activated with HATU (0.6 M, 4.3 eq) in DMF.

The peptide was cleaved from the resin using Reagent B cleavage cocktail (5 mL, 88% TFA, 5% phenol, 5% $H_2O$, 2% TIS) for 2 h at RT with agitation and isolated as described. The peptide pellet was re-dissolved in MeOH:$H_2O$ (5:95) supplemented with FA (0.1%) and purified by preparative HPLC-MS using a linear solvent gradient of 5-98% MeOH (0.1% FA) in $H_2O$ (0.1% FA) over 18 min at a flow rate of 20 mL/min. Collected fractions were pooled and lyophilised to afford the required peptide as a yellow solid.

MS (TOF MS ES+), m/z: calculated for $C_{64}H_{90}N_{16}O_{19}$ $[M+2H]^{2+}$: 694.76, found: 694.80; Analytical RP-HPLC: Gradient: 5-98% MeOH (0.1% FA) in $H_2O$ (0.1% FA) over 18 min, RT = 7.27 min.

**FAM-KRAS**

The peptide was synthesised on pre-loaded *N*-Fmoc-Met-Wang resin. Fmoc-βAla-OH (0.6 M, 4.3 eq) in DMF was activated with HATU (0.6 M, 4.3 eq) in DMF. A solution of FAM (3 eq) in DMF was activated with HOBt (3 eq) and DIC (3 eq) and added to the resin-bound peptide. The mixture was heated by microwave irradiation at 60 °C for 20 min. The resin was washed with DCM, piperidine (20%) in DMF, and DMF.

The peptide was cleaved from the resin using Reagent K cleavage cocktail (5 mL, 82.5% TFA, 5% phenol, 5% $H_2O$, 5% thioanisole, 2.5% EDT) for 2 h at RT with agitation and isolated as described. The peptide pellet was re-dissolved in MeCN:$H_2O$ (5:95) supplemented with FA (0.1%) and purified by preparative HPLC-MS using a linear solvent gradient of 5-98% MeCN

(0.1% FA) in $H_2O$ (0.1% FA) over 18 min at a flow rate of 20 mL/min. Collected fractions were pooled and lyophilised to afford the required peptide as a yellow solid.

MS (TOF MS ES+), m/z: calculated for $C_{56}H7_8N_9O_{14}S_2$ $[M+H]^+$: 1164.51, found: 1164.51; Analytical RP-HPLC: Gradient: 5-98% MeCN (0.1% FA) in $H_2O$ (0.1% FA) over 18 min, RT = 8.22 min.

**Pal-GobX-TAMRA**

The peptide was synthesised on an Activo-P11 Automated Peptide Synthesizer (Activotec, UK) on Universal NovaTag resin (Merck). HATU was used for all coupling reactions. The pseudoproline dipeptide *N*($\alpha$)-Fmoc-Tyr($^t$Bu)-Ser($\psi^{Me,Me}$pro)-OH (Novabiochem) was used to prevent aggregation, and the piperidine solution was supplemented with FA (1%) to suppress aspartimide formation. The peptidyl-resin was heated during synthesis until incorporation of the *N*-Fmoc-Cys(*S*-Tmp)-OH amino acid.

The Tmp protecting group was selectively cleaved using a solution of DTT (5% (w/v)) in NMM (0.1 M) and DMF (5 mL) for 3 × 5 min. Palmitoyl chloride (20 eq) in DMF (10 mL) and DMAP (0.1 eq) dissolved in a minimal amount of pyridine were added to the resin (0.05 mmol) and incubated for 16 h at RT. The resin was filtered and washed with DMF and DCM. Subsequent removal of the MMT protecting group was achieved by treating the resin twice with HOBt (0.6 M, 10 mL) in DCM:TFE (1:1) for 1 h.

The peptide was TAMRA labelled using a solution of TAMRA (4 eq) in DMSO:NMP (1:1). DIPEA (4 eq) was added, followed by HOBt (1 M, 2 eq) in NMP. After 3 min, DIC (2 eq) was added, then after 30 min the solution was added to the resin and allowed to react overnight. The resin was washed with DMF then DCM.

The peptide was cleaved from the resin by addition of cleavage cocktail (10 mL, 92.5% TFA, 2.5% $H_2O$, 2.5% EDT, 2.5% TIS). After dissolving in MeOH the peptide was purified on a C8 reverse phase HPLC column (Agilent PrepHT Zorbax 300SB-C8, 21.2 × 250 mm, 7 µm) using a linear solvent gradient of 28-81% MeCN (0.08% TFA) in $H_2O$ (0.08% TFA) over 40 min at a flow rate of 8 mL/min. The peak fraction was analysed by LC–MS on an Agilent 1100 LC-MSD. Collected fractions were pooled and lyophilised to afford the required peptide as a purple solid.

MS (TOF MS ES+), m/z: calculated for $C_{151}H_{217}N_{34}O_{35}S$ $[M+H]^+$: 3098.60, found: 3098.16; Analytical RP-HPLC: Gradient: 5-90% MeCN (0.08% TFA) in $H_2O$ (0.08% TFA) over 8.5 min, RT = 8.0 min.

## Biological and Biochemical Methods

### General Information

HEK-293a cells stably transfected with *SHH* (HEK-293 $SHH^+$ cells) were maintained as previously described in DMEM supplemented with 10% FBS (DMEM/FBS).[6] SHH-Light2 cells were a generous gift from Prof. James K. Chen (Stanford University, USA) and were maintained as previously described in high glucose- and sodium pyruvate-containing DMEM containing G418 (400 µg/mL) and Zeocin (150 µg/mL), and supplemented with 10% iron fortified calf serum (DMEM/FCS).[6] NMT1 was purified as previously described.[7] APT1 and APT2 expression plasmids were a generous gift from Dr Matthew Child (Imperial College, UK) and Prof. Matthew Bogyo (Stanford University, USA). The SCREEN-WELL® FDA-approved drug library (BML-2843, Enzo) was a generous gift from Dr Vania Braga (Imperial College, UK) and was stored at -80 °C. Trypsin (Promega), FTase (Jena Bioscience) and GGTase (Jena Bioscience) were stored at -80 °C. Pal-CoA (Sigma Aldrich) was stored in NaOAc (0.1 M, pH 6.0) at -80 °C.

Assay data was evaluated using Microsoft Excel 2013 and GraphPad Prism 5.0. Fluorescence anisotropy was calculated using equation 1 and fluorescence intensity calculated using equation 2.

$$(1)\ FA = \frac{S - GP}{S + 2GP}$$

$$(2)\ FI = S + 2GP$$

Where S is signal in the S-channel, P is signal in the P-channel, and G is the G-factor. Assay characteristics were calculated using equations 3-5.[8]

$$(3)\ S/B = \frac{\mu_p}{\mu_n}$$

$$(4)\ S/N = \frac{\mu_p - \mu_n}{\sigma_n}$$

$$(5)\ Z' = 1 - \frac{3(\sigma_p + \sigma_n)}{|\mu_p - \mu_n|}$$

Where µ is the sample mean, σ is the sample standard deviation, and p and n subscripts denote positive and negative controls, respectively.

Mixtures described as % represent v/v unless otherwise stated.

## Recombinant Protein Production

### HHAT-P100(sol)

HHAT-enriched solubilised membrane fractions (HHAT-P100(sol)) were prepared as previously described,[4] with the following modifications. HEK293a cells overexpressing HHAT-FLAG-His (HEK293a HHAT$^{+}$) were cultured, harvested by trypsinisation, and washed with PBS as previously described.[9] Cells (5 × $10^{6}$) in DMEM/FBS (35 mL) were inoculated on sterilised CultiSphere S microcarriers (100 mg) in a spinner flask (Corning), and incubated as previously described. After 24 h, DMEM/FBS (15 mL) was added and cells incubated with stirring (~30 rpm), with addition on DMEM/FBS (50 mL) after 24 h. DMEM/FBS (50 mL) was refreshed every subsequent 24 h. After 5 days, microcarriers were collected by centrifugation (1,000 g, 1 min), and washed with PBS (25 mL). Microcarriers were digested with trypsin (3 mL) with visual monitoring by microscopy (5-15 min, see Supplementary Figure S11D) to release HEK293a HHAT$^{+}$ cells, which were washed with DMEM/FBS (25 mL) and PBS (25 mL). Cells were lysed by manual homogenisation (30 strokes in a Dounce homogenizer with a tight fitting pestle) and immediately centrifuged (100,000 g, 1 h, 4 °C) to generate a P100 fraction that was solubilised and enriched in HHAT through Ni-NTA purification as previously described.[4]

### Purified HHAT

Full-length human HHAT (UniProt Q5VTY9) C-terminally fused with a 1D4 epitope tag[10](Oprian, 1987 #61) was cloned into the pHL vector,[11] expressed by transient transfection in HEK293S-GnTI- (ATCC CRL-3022) cells and purified via antibody purification with Rho-1D4 antibody (University of British Columbia) coupled to CNBr-activated sepharose beads (GE Healthcare) followed by size exclusion, as previously described.[12] Purified HHAT was concentrated to 1-2 mg/mL in storage buffer (10 mM HEPES, 150 mM NaCl, 5% glycerol, 0.025% (w/v) DDM, 0.0006% (w/v) cholesterol-hemisuccinate, pH 7.5) and flash-frozen in liquid nitrogen.

### Purified SHH(FL)

The human Sonic hedgehog N-terminal signalling domain (SHH(FL), residues 24-193, UniProt Q15465) fused to a C-terminal 3C protease cleavable hexahistidine ($His_6$)-tag was cloned into the pET22b expression vector (Novagen) and expressed in *E. coli* Rosetta 2(DE3)pLysS cells following previously established procedures.[13] In short, cultures were grown at 37 °C to an $OD_{600}$ of 0.8, cooled to 18 °C, induced with isopropyl-β-D-thiogalactopyranoside (IPTG, 0.2 mM) and then grown for ~16 h before harvesting. The pellet was resuspended in lysis buffer (10 mM sodium phosphate, 500 mM NaCl, 1 mM β-mercaptoethanol, pH 7.4) and EDTA-free protease inhibitor cocktail (Roche). Cells were broken using sonication (Sonics Materials™, VCX 500) and fractionated by centrifugation (30,000 g, 45 min, 5 °C). The supernatant was incubated with TALON® beads for 1 h at RT. Beads were stepwise washed with lysis buffer containing 0, 5 and 7.5 mM imidazole. SHH(FL) was eluted in lysis buffer containing 250 mM imidazole. 3C protease (1:100 molar ratio) was added to eluted SHH(FL) and dialysed overnight. SHH(FL) was subsequently purified via size exclusion

chromatography using a Superdex 75 column (GE Healthcare) equilibrated in 5 mM sodium phosphate, 150 mM NaCl, 0.5 mM DTT, pH 5.5.

**APT1 and APT2**

Full-length human APT1 (residues 1-230, Uniprot O75608) and full-length human APT2 (residues 1-231, UniProt O95372) were produced as previously described,[14] with the following modifications. Constructs in pET28a were transformed into chemically competent *E. coli* BL21(DE3)pLysS (Sigma). Single colonies were inoculated into LB (5 mL) supplemented with kanamycin (50 µg/mL) and chloramphenicol (25 µg/mL; LB-kan/cam) and cells grown overnight at 37 °C with shaking (180 rpm). The overnight culture (2 mL) was used to inoculate LB-kan/cam (2 L) and cells grown to an $OD_{600}$ of 0.6 (4-5 h) before expression was induced with IPTG (1 mM) for 4 h at 37 °C with shaking (180 rpm). Cells were harvested via centrifugation (2,000 g, 20 min, 4 °C). Cell pellets were resuspended in lysis buffer (50 mM TRIS, 500 mM NaCl, 10% glycerol, 15 mM imidazole, pH 8.8) and lysed via one passage at 25 kPSI through a Cell Disruptor (Constant Systems). Lysates were cleared by centrifugation (20,000 g, 30 min, 4 °C), and the supernatant passed through a HisTrap™ Fast Flow column (GE Healthcare). Bound protein was eluted with elution buffer (50 mM TRIS, 150 mM NaCl, 300 mM imidazole, pH 8.8), and concentrated to 2 mL in a Protein Concentrator PES (Pierce, 10,000 MWCO). The sample was cleared by centrifugation (20,000 g, 5 min, 4 °C) and purified using a S75 16/600 gel filtration column (Generon), eluting with storage buffer (50 mM TRIS, 150 mM NaCl, pH 7.4). Fractions containing protein were pooled, concentrated (5 mg/mL APT1, 4 mg/mL APT2), flash frozen, and stored at −80 °C.

**Fluorescence Anisotropy**

Fluorescence measurements were recorded on an EnVision Xcite 2104 (PerkinElmer).

FAM-labelled peptides were measured using a FITC FP D505fp mirror module, FITC FP 480 nm excitation filter (30 nm bandwidth), FITC FP P-pol 535 nm first emission filter (40 nm bandwidth), and FITC FP S-pol 535 nm second emission filter (40 nm bandwidth). Continuous reaction monitoring was conducted using measurement height = 9.52 mm, 8 flashes/well, PMT gain = 319, and G-factor = 1.09. For the HHAT pilot screen, stopped-assay measurements were performed using measurement height = 9.52 mm, 60 flashes/well, PMT gain = 319, and G-factor = 1.09. The NMT acyl-cLIP assay continuous reaction monitoring was conducted using measurement height = 6.6 mm, 1 flash/well, PMT gain = 163, and G-factor = 1.05

TAMRA-labelled peptides were measured using a BODIPY TMR FP D555fp mirror module, BODIPY TMR FP 531 nm excitation filter (25 nm bandwidth), BODIPY TMR FP P-pol 579 nm first emission filter (25 nm bandwidth), and BODIPY TMR FP S-pol 579 nm second emission filter (25 nm bandwidth). Continuous reaction monitoring was conducted using measurement height = 6.5 mm, 200 flashes/well, PMT gain = 255, and G-factor = 1.6.

**HHAT Acyl-cLIP Assay**

HHAT-P100(sol) (450 µg/mL) in solubilisation buffer (20 mM HEPES, 350 mM NaCl, 1% (w/v) DDM, 5% glycerol, pH 7.3) was diluted 1:10 in SHH-FAM (2 µM) in reaction buffer (100 mM MES, 20 mM NaCl, 1 mM DTT, 1 mM TCEP, 0.1% (w/v) BSA, pH 6.5). The HHAT/SHH-FAM solution (12 µL/well) was dispensed into a black 384-well plate (Corning, 3575). The reaction was initiated by addition of Pal-CoA (7.5 µM) in reaction buffer (8 µL/well), and fluorescence readings collected every 1 min for 1 h. Under stopped-assay conditions, the reaction was halted by addition of SHH (220 µM) in reaction buffer (2 µL).

Detergents (2 mM) or BSA (0.15 mM) were tested for generation of polarised fluorescence from Pal-SHH-FAM or SHH-FAM (0.1 µM) in 100 mM MES, 20 mM NaCl. Detergent and BSA titrations were performed using 2-fold serial dilutions.

Small molecule inhibitors in DMSO were dispensed using an Echo 550 liquid handler (Labcyte), with a DMSO backfill volume of 50 nL. Substrate competitors SHH, SHH(FL), or Pal-SHH were serially diluted in reaction buffer (1:3) prior to addition to the HHAT/SHH-FAM solution. Initial rate constants were determined by linear regression, normalised to DMSO negative controls and background corrected to positive controls with solubilisation buffer instead of HHAT. Heat-inactivated HHAT-P100(sol) controls were heated (95 °C, 5 min), cooled on ice and briefly centrifuged to collect the condensate.

The pilot HTS used purified HHAT (1 µM), which was diluted 1:10 in SHH-FAM (2 µM) in reaction buffer and assays performed under stopped-assay conditions as previously described.

**NMT Acyl-cLIP Assay**

SRC-FAM (2 µM) was prepared in reaction buffer (20 mM potassium phosphate, 0.5 mM EDTA, 0.1% Triton X-100, pH 7.9–8.0) and the solution (5 µL/well) was dispensed into a low-volume black 384-well plate (Corning, 4514). Myristoyl-CoA (20 µM) in reaction buffer (2 µL/well) was added, and the reaction initiated by addition of NMT1 (220 nM) in reaction buffer (3 µL/well) or reaction buffer (3 µL/well) only. Fluorescence readings were collected every 1 min for 1 h.

**FTase/GGTase Prenyl-cLIP Assay**

FAM-KRAS (2 µM) was prepared in reaction buffer (50 mM TRIS, 20 mM KCl, 1 mM DTT, 50 µM $ZnCl_2$, 0.1% (w/v) DDM, pH 7.5) with either farnesyl pyrophosphate (8 µM) or geranylgeranyl pyrophosphate (8 µM). The FAM-KRAS/prenyl pyrophosphate solution (10 µL/well) was dispensed into a black 384-well plate (Corning, 3575), and the reaction initiated by the addition of FTase (50 nM), GGTase (50 nM) in reaction buffer (10 µL/well), or reaction buffer (10 µL/well) only. Fluorescence readings were collected every 1 min for 1 h.

**Trypsin Deacyl-cLIP Assay**

Pal-SHH-FAM (1 µM) in PBS with 0.1% (w/v) DDM (10 µL/well) was dispensed into a black 384-well plate (Corning, 3575), and the reaction initiated by addition of trypsin (8 nM) in PBS

with 0.1% (w/v) DDM, or PBS with 0.1% (w/v) DDM (10 µL/well) only. Fluorescence readings were collected every 1 min for 1 h.

### APT1/2 Deacyl-cLIP Assay

APT1 or APT2 (400 nM) in reaction buffer (100 mM MES, 1 mM DTT, 1 mM TCEP, 0.1% (w/v) DDM, pH 6.5) was dispensed into a black 384-well plate (Corning, 3575), and the reaction initiated by addition of Pal-GobX-TAMRA (1 µM) in reaction buffer. Fluorescence readings were collected every 1 min for 1 h.

### SHH-Light2 Signalling Assay

HEK293a SHH$^+$ cells (120,000 cells/well) were plated in a 12-well cell culture treated plate (Nunc) in DMEM/FBS (1 mL/well) and cultured for 24 h. Cells were washed with PBS, followed by the addition of DMEM/FBS (1 mL) containing DMSO (0.2%) or varying inhibitor concentrations in DMSO (0.041-30 µM). SHH-Light2 cells (20,000 cells/well) were plated in a 96-well plate in DMEM/FCS (50 µL/well). After 24 h, conditioned medium (300 µL) was removed from each well of the HEK293a SHH$^+$ cells, centrifuged (1,000 g, 10 min), and added to three wells of SHH-Light2 cells (100 µL/well, 150 µL final volume). SHH-Light2 cells were cultured for a further 48 h, and washed with PBS prior to analysis. *Firefly* and *Renilla* luciferase activity was recorded using the Dual-Luciferase reporter system (Promega Corporation, USA). Passive lysis buffer (20 µL) was added to each well and cells incubated for 30 min at RT. The lysate (5 µL) was transferred to a white opaque 96-well plate (Corning, 3362). Luciferase Assay Reagent II (20 µL/well) was added and the luminescence immediately recorded using a SpectraMax i3x spectrophotometer (Molecular Devices LLC). Stop&Glo (1x) substrate in Stop&Glo buffer (20 µL/well) was added and the luminescence immediately recorded. *Firefly* luciferase activity was normalised to DMSO vehicle and background corrected to unconditioned media.

### In-Cell YnC$_{15}$ Tagging Assay

HEK293a SHH$^+$ cells (500,000 cells/well) were plated in a 6-well plate in DMEM/FBS (3 mL/well) and cultured for 24 h. Cells were treated with inhibitors in DMSO (20 µM), two RUSKI-201 (20 µM) blind-positive controls and two DMSO (7.5 µL) blind-negative controls. After 1 h, YnC$_{15}$ (3 µL, 20 mM in DMSO) was added and cells cultured for a further 6 h before washing with PBS. Cells were lysed in PBS with Triton X-100 (1%), SDS (0.1% (w/v)) and Complete EDTA-free protease inhibitor cocktail (Roche Diagnostics). Cells were removed from plates by scraping, and lysates centrifuged (13,000 g, 10 min, 4 °C). The protein concentration of the supernatant was estimated using the DC Protein Assay (Bio-Rad) and adjusted to 1 mg/mL with lysis buffer. AzTB (1 µL, 10 mM in DMSO), $CuSO_4$ (2 µL, 50 mM in $H_2O$), TCEP (2 µL, 50 mM in $H_2O$) and TBTA (1 µL, 10 mM in DMSO) were combined, and this mixture (6 µL) added to the lysate (100 µL). Samples were shaken at RT for 1 h, then proteins precipitated by addition of methanol (200 µL), chloroform (50 µL) and water (100 µL) followed by centrifugation (13,000 g, 5 min, 4 °C). The upper aqueous layer was removed without disturbing the protein layer at the interphase, methanol (0.5 mL) was added, and the precipitated proteins isolated by centrifugation (13,000 g, 10 min, 4 °C). Protein pellets were

washed twice with methanol, briefly air-dried and re-dissolved in PBS containing SDS (0.2% (w/v)) and DTT (0.1 mM) using sonication to a final concentration of 1 mg/mL.

10 µg of protein were separated on a 15% SDS-polyacrylamide gel. Fluorescence was recorded using a Typhoon imager (GE Healthcare; excitation laser: 532 nm, emission filter: LPG (575-700 nm); PMT: 750 V).

Proteins were transferred to a nitrocellulose membrane by wet transfer (100 V, 1 h), and membranes blocked with BSA (5% (w/v)) in TBS-T (50 mM TRIS, 150 mM NaCl, 0.1% Tween-20, pH 7.5) for 1 h at RT, followed by overnight incubation at 4°C with rabbit α-SHH (Santa Cruz Biotechnology; sc-9024; 1:200) in TBS-T with BSA (0.5% (w/v)) or mouse α-Tubulin (Santa Cruz Biotechnology; sc-8035; 1:200) in TBS-T with BSA (0.5% (w/v)). Membranes were washed with TBS-T (4 × 5 min) and incubated at RT for 1 h with HRP-conjugated Goat-α-mouse IgG (Advansta; 1:10,000) in TBS-T with BSA (0.5% (w/v)) or Goat-α-rabbit IgG (Advansta; 1:10,000) in TBS-T with BSA (0.5% (w/v)). Membranes were washed with TBS-T (4 × 5 min) and developed with Luminata Crescendo Western HRP substrate (Millipore) on a Fujifilm LAS-3000 imager. $YnC_{15}$-labelled SHH band intensities were quantified using ImageJ 1.50i (National Institute of Health, USA).

**MTS Assay**

HEK293a $SHH^{+}$ cells (5,000 cells/well) were plated in a 96-well plate in DMEM/FBS (50 µL/well) and cultured for 24 h. The medium was exchanged for DMEM/FBS (100 µL/well) supplemented with either DMSO (1 µL), Puromycin (2 µg/mL), or inhibitors (0.14-100 µM) and cells incubated for 72 h. of A mixture of 1-methoxy phenazine methosulfate (PMS, 0.92 mg/mL) and MTS (2 mg/mL) in PBS (20 µL/well) was added and plates incubated for 3 h at 37 °C. The absorption at 490 nm was recorded using an EnVision Xcite 2104 and responses normalised to vehicle control and background corrected to Puromycin-treated samples.

## References


[1] A. Stetsenko, A. Guskov, *Crystals* **2017**, *7*, 197.

[2] W. P. Heal, B. Jovanovic, S. Bessin, M. H. Wright, A. I. Magee, E. W. Tate, *Chem. Commun. Camb. Engl.* **2011**, *47*, 4081–4083.

[3] A. D. Konitsiotis, B. Jovanović, P. Ciepla, M. Spitaler, T. Lanyon-Hogg, E. W. Tate, A. I. Magee, *J. Biol. Chem.* **2015**, *290*, 3293–3307.

[4] T. Lanyon-Hogg, N. V. Patel, M. Ritzefeld, K. J. Boxall, R. Burke, J. Blagg, A. I. Magee, E. W. Tate, *SLAS Discov.* **2017**, *22*, 418–424.

[5] T. Lanyon-Hogg, N. Masumoto, G. Bodakh, A. D. Konitsiotis, E. Thinon, U. R. Rodgers, R. J. Owens, A. I. Magee, E. W. Tate, *Data Brief* **2016**, *7*, 257–281.

[6] U. R. Rodgers, T. Lanyon-Hogg, N. Masumoto, M. Ritzefeld, R. Burke, J. Blagg, A. I. Magee, E. W. Tate, *ACS Chem. Biol.* **2016**, *11*, 3256–3262.

[7] V. Goncalves, J. A. Brannigan, E. Thinon, T. O. Olaleye, R. Serwa, S. Lanzarone, A. J. Wilkinson, E. W. Tate, R. J. Leatherbarrow, *Anal. Biochem.* **2012**, *421*, 342–344.

[8] J. H. Zhang, T. D. Chung, K. R. Oldenburg, *J. Biomol. Screen.* **1999**, *4*, 67–73.

[9] T. Lanyon-Hogg, N. Masumoto, G. Bodakh, A. D. Konitsiotis, E. Thinon, U. R. Rodgers, R. J. Owens, A. I. Magee, E. W. Tate, *Anal. Biochem.* **2015**, *490*, 66–72.

[10] R. S. Molday, D. MacKenzie, *Biochemistry (Mosc.)* **1983**, *22*, 653–660.

[11] A. R. Aricescu, W. Lu, E. Y. Jones, *Acta Crystallogr. D Biol. Crystallogr.* **2006**, *62*, 1243–1250.

[12] E. F. X. Byrne, R. Sircar, P. S. Miller, G. Hedger, G. Luchetti, S. Nachtergaele, M. D. Tully, L. Mydock-McGrane, D. F. Covey, R. P. Rambo, et al., *Nature* **2016**, *535*, 517–522.

[13] B. Bishop, A. R. Aricescu, K. Harlos, C. A. O'Callaghan, E. Y. Jones, C. Siebold, *Nat. Struct. Mol. Biol.* **2009**, *16*, 698–703.

[14] M. Garland, C. J. Schulze, I. T. Foe, W. A. van der Linden, M. A. Child, M. Bogyo, *PloS One* **2018**, *13*, e0190255.
